# Effects of Two Moderate-Intensity Aerobic Exercise Prescriptions on Inflammatory Cytokines and Oxidative Stress Biomarkers in Obese Hispanic Females

**DOI:** 10.3390/ijms27041834

**Published:** 2026-02-14

**Authors:** Kyung-Shin Park, Paola Canales Gonzalez, Miguel Nieto, Brett S. Nickerson

**Affiliations:** 1 Department of Health Sciences, Texas A&M International University, Laredo, TX 78041, USAmiguelnieto@dusty.tamiu.edu (M.N.); 2 School of Health and Rehabilitation Sciences, Ohio State University, Columbus, OH 43210, USA; brett.nickerson@osumc.edu

**Keywords:** inflammatory cytokines, oxidative stress, obesity, aerobic exercise

## Abstract

This study examined effects of two moderate-intensity aerobic exercise prescriptions on inflammatory cytokines and oxidative stress biomarkers in middle-aged obese Hispanic females. Fifty-four subjects were randomly assigned to a lower-moderate intensity group (55% VO_2_max, LT, n = 18), an upper-moderate intensity group (70% VO_2_max, HT, n = 19), or a non-exercise control group (CON, n = 17). Blood samples collected before and after a 12-week intervention were analyzed for tumor necrosis factor-alpha (TNF-α), adiponectin, C-reactive protein (CRP), total antioxidant status (TAS), and 8-hydroxy-2′-deoxyguanosine (8-OHdG). Body fat percentage (%BF) and visceral adipose tissue (VAT) were assessed using dual-energy X-ray absorptiometry. TNF-α significantly decreased in both LT (*p* = 0.004) and HT (*p* < 0.001). Significant increases in adiponectin (*p* = 0.001) and reductions in CRP (*p* < 0.001) were observed within the HT, whereas these changes were not significant within the LT. TAS significantly increased in both exercise groups (*p* < 0.001), and 8-OHdG significantly decreased in the HT (*p* < 0.001) and LT (*p* = 0.002). Both LT and HT demonstrated significant reductions in %BF (*p* < 0.001) and VAT (*p* < 0.05), with no significant changes in CON. Results indicate that moderate-intensity aerobic exercise improves inflammatory and oxidative stress profiles when total exercise volume is matched, regardless of differences in exercise intensity within the moderate range. Although post-intervention differences between groups were not statistically significant, the observation that CRP, adiponectin, and 8-OHdG changed significantly only within HT suggests that exercise intensity may influence biomarker responsiveness and warrants further investigation.

## 1. Introduction

Obesity is a worldwide issue characterized by excessive accumulation of adipose tissue and increased body weight. In the United States, the Hispanic community has one of the highest rates of obesity, with 26.6% of Hispanic children between the ages of two and nineteen being obese, and 45.6% of Hispanic adults aged twenty and over affected by obesity [1]. This condition has been strongly associated with the development of multiple comorbidities, including metabolic syndrome, type 2 diabetes, cancer, hypertension, and cardiovascular diseases. High levels of visceral adipose tissue (VAT) and elevated fat mass are known to contribute to increased production of pro-inflammatory cytokines such as tumor necrosis factor-alpha (TNF-α), interleukin-6 (IL-6), and C-reactive protein (CRP), which generate reactive oxygen species (ROS) and promote oxidative stress (OS). These factors collectively trigger chronic, low-grade systemic inflammation, which plays a critical role in the pathogenesis of various metabolic and cardiovascular disorders [2,3,4].

It is generally accepted that regular physical activity has therapeutic effects on systemic inflammation and redox homeostasis. Numerous studies have demonstrated that chronic aerobic exercise can reduce circulating levels of pro-inflammatory cytokines and improve the body’s antioxidant capacity [5,6,7,8]. In particular, muscle-derived IL-6 and exercise-induced increases in catecholamines such as epinephrine have been shown to inhibit TNF-α expression and support anti-inflammatory signaling pathways [9,10]. Furthermore, exercise promotes enhanced oxygen consumption and ROS generation, which stimulate endogenous antioxidant defense systems and enhance redox adaptation [11].

While the anti-inflammatory effects of exercise are well documented, there is also compelling evidence that reductions in body fat, especially VAT, contribute significantly to reductions in systemic inflammation and oxidative stress. Studies have shown that increased adiposity correlates positively with TNF-α and OS markers and negatively with antioxidant levels, independent of age or diet [12,13,14,15]. Reductions in VAT through lifestyle interventions, including exercise, are associated with decreased levels of TNF-α and CRP, and increased levels of adiponectin and antioxidant biomarkers [16,17,18].

Although there is substantial evidence supporting the role of aerobic exercise in modulating inflammatory cytokines and oxidative stress, much of this research does not clearly distinguish the effects of exercise intensity from those of total exercise dose or volume. Few studies have systematically evaluated the chronic effects of different aerobic exercise intensity prescriptions under volume-matched conditions on adipocytokines and oxidative stress biomarkers, particularly in underrepresented populations such as Hispanic females. Given the disproportionately high rates of obesity and metabolic disease in this group, there is a critical need to investigate how aerobic exercise performed at different intensity levels within the moderate range, when total exercise volume is controlled, influences inflammation and oxidative stress. Therefore, the present study aims to examine the changes in pro- and anti-inflammatory cytokines and oxidative stress biomarkers in response to 12 weeks of volume-matched moderate-intensity aerobic exercise performed at two different intensity prescriptions in obese Hispanic females.

## 2. Results

### 2.1. Physical Characteristics

Before the 12-week intervention, the three groups (CON, HT, LT) did not differ significantly in any baseline physical characteristics, including age, height, body weight, BMI (body mass index), body fat percentage, or visceral adipose tissue (multivariate Pillai’s trace = 0.006, F(8,98) = 0.04, *p* = 1.000; all univariate *p* ≥ 0.345), indicating that groups were equivalent prior to the 12-week intervention. The detailed baseline characteristics of the subjects are presented in Table 1.

### 2.2. Changes in Body Composition Variables

A repeated-measures MANOVA including the four body composition variables revealed a significant multivariate main effect of Time (Pillai’s trace = 0.785, F(4,48) = 43.74, *p* < 0.001, partial η^2^ = 0.785), indicating substantial pre-to-post changes across the sample. The multivariate main effect of Group was not significant (Pillai’s trace = 0.074, F(8,98) = 0.47, *p* = 0.875, partial η^2^ = 0.037), but the Time × Group interaction was significant (Pillai’s trace = 0.869, F(8,98) = 9.42, *p* < 0.001, partial η^2^ = 0.435), indicating group-specific patterns of change.

Univariate analyses revealed significant main effects of Time for body weight (F(1,51) = 58.14, *p* < 0.001), BMI (F(1,51) = 59.35, *p* < 0.001), body fat percentage (F(1,51) = 171.53, *p* < 0.001), and VAT (F(1,51) = 28.28, *p* < 0.001). Time × Group interactions were also significant for body weight (F(2,51) = 16.03, *p* < 0.001), BMI (F(2,51) = 16.29, *p* < 0.001), body fat percentage (F(2,51) = 58.70, *p* < 0.001), and VAT (F(2,51) = 14.74, *p* < 0.001).

Post hoc pairwise comparisons showed no significant pre–post changes in the control group for any variable (all *p* ≥ 0.345). In contrast, both exercise groups showed significant reductions in variables as shown in Table 2. In the HT group, body weight decreased (Δ = −3.64 kg, *p* < 0.001), BMI decreased (Δ = −1.37 kg·m^−2^, *p* < 0.001), body fat percentage decreased (Δ = −4.97%, *p* < 0.001), and VAT decreased (Δ = −0.27 kg, *p* < 0.001). In the LT group, body weight decreased (Δ = −2.31 kg, *p* < 0.001), BMI decreased (Δ = −0.87 kg·m^−2^, *p* < 0.001), body fat percentage decreased (Δ = −3.41%, *p* < 0.001), and VAT decreased (Δ = −0.08 kg, *p* = 0.037).

Between-group comparisons at post-intervention showed no significant multivariate differences (Pillai’s trace = 0.241, F(8,98) = 1.68, *p* = 0.114). Univariate analyses indicated that only body fat percentage differed significantly among groups, with HT showing a lower post-intervention value compared with CON (mean difference = −5.37%, *p* = 0.006); all other variables showed no significant between-group differences (all *p* ≥ 0.052).

### 2.3. Inflammatory Cytokines and Oxidative Stress Markers

At baseline, no significant group differences were observed for TNF-α (*p* = 0.942), CRP (*p* = 0.917), adiponectin (*p* = 0.346), 8-hydroxy-2′-deoxyguanosine (8-OHdG, *p* = 0.965), or total antioxidant status (TAS, *p* = 0.944), indicating that all groups were comparable prior to the 12-week intervention.

A repeated-measures MANOVA including five biomarkers (TNF-α, CRP, adiponectin, 8-OHdG, TAS) revealed a significant multivariate main effect of Time (Pillai’s trace = 0.465, F(5,47) = 8.17, *p* < 0.001, partial η^2^ = 0.465), indicating overall pre-to-post changes across subjects. The multivariate main effect of Group was not significant (Pillai’s trace = 0.092, F(10,96) = 0.46, *p* = 0.911, partial η^2^ = 0.046), whereas the Time × Group interaction was significant (Pillai’s trace = 0.505, F(10,96) = 3.24, *p* = 0.001, partial η^2^ = 0.253), suggesting that biomarker changes over time, differing by group.

Univariate analyses demonstrated significant main effects of Time for all biomarkers, including TNF-α (F(1,51) = 17.19, *p* < 0.001), CRP (F(1,51) = 16.85, *p* < 0.001), adiponectin (F(1,51) = 6.34, *p* = 0.015), 8-OHdG (F(1,51) = 18.94, *p* < 0.001), and TAS (F(1,51) = 15.83, *p* < 0.001). Time × Group interactions were also significant for TNF-α (F(2,51) = 6.45, *p* = 0.003), CRP (F(2,51) = 9.46, *p* < 0.001), adiponectin (F(2,51) = 3.35, *p* = 0.043), 8-OHdG (F(2,51) = 6.73, *p* = 0.003), and TAS (F(2,51) = 5.92, *p* = 0.005).

**Figure 1:**
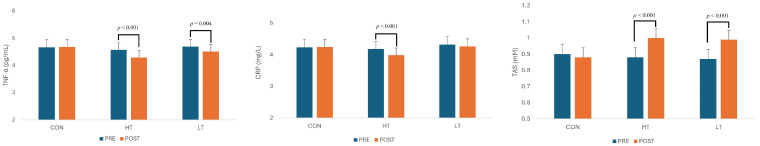
Changes of inflammatory cytokines at baseline (PRE) and after 12 weeks of aerobic exercise intervention (POST) at two moderate-intensity prescriptions. Values are means ± SE. CON: Control group; HT: Upper-moderate intensity group (70% VO_2_max); LT: Lower-moderate intensity group (55% VO_2_max); TNF-α: Tumor necrosis factor alpha; CRP: C-reactive protein. Significant within-group changes are shown where applicable. Post-intervention between-group differences were not statistically significant.

**Figure 2:**
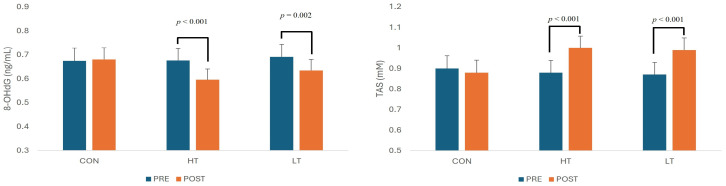
Changes of oxidative stress markers at baseline (PRE) and after 12 weeks of aerobic exercise intervention (POST) at two moderate-intensity prescriptions. Values are means ± SE. CON: Control group; HT: Upper-moderate intensity group (70% VO_2_max); LT: Lower-moderate intensity group (55% VO_2_max); TAS: Total antioxidant status; 8-OHdG: 8-hydroxydeoxyguanosine. Significant within-group changes are shown where applicable. Post-intervention between-group differences were not statistically significant.

Post hoc pairwise comparisons indicated no significant pre–post changes in the control group for any biomarker (all *p* > 0.59). In contrast, both exercise groups demonstrated significant changes at post. Changes in inflammatory cytokines and markers of oxidative stress are presented in Figure 1 and Figure 2. The HT group exhibited significant reductions in TNF-α (Δ = −0.281, *p* < 0.001), CRP (Δ = −0.205, *p* < 0.001), and 8-OHdG (Δ = −0.080, *p* < 0.001), alongside significant increases in adiponectin (Δ = +0.115, *p* = 0.001) and TAS (Δ = +0.123, *p* < 0.001). The LT group also demonstrated significant decreases in TNF-α (Δ = −0.182, *p* = 0.004) and 8-OHdG (Δ = −0.057, *p* = 0.002), along with an increase in TAS (Δ = +0.123, *p* < 0.001), though to a lesser extent. However, unlike HT, the LT group showed no significant change in adiponectin (*p* = 0.227) or CRP (*p* = 0.100).

Importantly, one-way ANOVA of post-intervention values revealed no statistically significant between-group differences for TNF-α, CRP, adiponectin, 8-OHdG, or TAS (all *p* ≥ 0.22). Accordingly, although within-group responses differed across exercise prescriptions, post-intervention biomarker levels did not differ significantly between LT and HT.

## 3. Discussion

The present study examined effects of two moderate-intensity aerobic exercise prescriptions on pro- and anti-inflammatory cytokines, as well as biomarkers of oxidative stress, in obese Hispanic females. Following the 12-week intervention, significant reductions in TNF-α were observed in both exercise groups. However, significant changes in adiponectin and CRP were found only in the upper-moderate intensity group. Greater reductions in body fat were also observed within this group. Both exercise groups showed significant improvements in oxidative stress and total antioxidant status, with no significant differences between the upper-moderate high intensity and lower-moderate intensity groups, even though greater reductions in body fat were observed within the HT group. No changes were observed in the control group.

Obesity is commonly associated with chronic inflammation in adipose tissue, which serves as a source of pro-inflammatory cytokines and reactive oxygen species [4]. Numerous studies have demonstrated a positive association between increased adiposity and elevated levels of pro-inflammatory cytokines and oxidative stress markers [13,19]. Conversely, increased body mass index has been negatively correlated with circulating adiponectin levels and total antioxidant capacity [20]. Accordingly, reductions in adipose tissue are frequently linked with decreases in oxidative stress and pro-inflammatory cytokine activity [21].

It is generally accepted that regular physical activity and exercise training reduce inflammatory cytokine activity and oxidative stress, while also enhancing antioxidant capacity. Acute bouts of aerobic exercise often trigger a temporary inflammatory response, marked by increased levels of pro-inflammatory cytokines such as TNF-α, CRP, and IL-6. These sessions also stimulate the production of ROS but simultaneously upregulate antioxidant defenses [22]. In contrast, long-term aerobic exercise training has been shown to decrease concentrations of inflammatory markers like TNF-α and CRP, while increasing levels of adiponectin, which promotes an anti-inflammatory state [6,8]. Moreover, regular training appears to induce physiological adaptations that both elevate antioxidant levels and suppress ROS production [5]. Despite this evidence, the specific effects of exercise intensity on inflammatory cytokines and oxidative stress remain inconclusive.

In the present study, TNF-α was significantly reduced in both exercise groups, whereas significant reduction in CRP was observed only within the HT group. Haijzadeh Maleki et al. [7] reported significant modulations in pro-inflammatory cytokines, including TNF-α, following 12 and 24 weeks of moderate-intensity aerobic training, suggesting that repeated exercise sessions may reduce the production of pro-inflammatory cytokines. It has also been suggested that regular exercise training lowers resting levels of pro-inflammatory cytokines through the upregulation of anti-inflammatory cytokines throughout the body [7]. Similarly, Koh and Park [23] reported significant reductions in TNF-α following a 4-week moderate-intensity aerobic intervention despite no accompanying weight loss. In contrast, CRP did not change significantly in that study, leading the authors to suggest that a longer intervention duration or a moderate degree of weight loss may be required to elicit changes in CRP levels. Consistent with this interpretation, other studies have suggested that reductions in fat mass play a central role in lowering CRP levels, with greater CRP reductions observed when exercise is accompanied by weight loss compared with exercise-only or weight-loss-only interventions [24,25].

Adiponectin increased significantly only in the HT group. Previous studies suggest that both intervention duration and the magnitude of weight loss may be important determinants of adiponectin responses. Koh and Park [23] reported no change in adiponectin following a short-term aerobic intervention without weight loss, whereas Mujumdar et al. [26] demonstrated significant increases in adiponectin after a 6-month progressive walking program that resulted in significant reductions in body weight and BMI in female participants. Similarly, a 12-week aerobic training program performed at 60–70% HRmax combined with a structured dietary regimen led to significant increases in adiponectin alongside substantial weight loss [27]. Collectively, these findings suggest that a threshold level of weight or fat mass reduction may be necessary to induce increases in adiponectin.

HT group experienced greater within-group reductions in body weight, body fat percentage, and VAT compared with the LT group, which may partially explain the significant within-group reductions in CRP and increases in adiponectin observed only in HT. However, because post-intervention between-group differences were not statistically significant, these findings should not be interpreted as evidence of definitive intensity-dependent superiority. Rather, they suggest that reductions in adiposity, rather than exercise intensity per se, likely played a substantial role in mediating these biomarker responses. Further studies incorporating larger sample sizes, dietary control, and direct mechanistic measures are warranted to clarify the relative contributions of exercise intensity and fat loss.

Both exercise prescriptions resulted in significant improvements in oxidative stress and antioxidant status, with no statistically significant between-group differences at post-intervention. Previous studies have consistently reported that chronic moderate- to moderately high-intensity aerobic exercise leads to reductions in oxidative stress biomarkers and concomitant increases in antioxidant capacity [7,28,29]. For example, Goto et al. [28] demonstrated that moderate-intensity aerobic exercise (50% VO_2_max) resulted in greater reductions in plasma 8-OHdG and other oxidative stress biomarkers compared with both lower (25% VO_2_max) and higher (75% VO_2_max) exercise intensities in healthy young adults. Similarly, Haijzadeh Maleki et al. [7] reported significant reductions in oxidative stress and increases in antioxidant biomarkers following 12 to 24 weeks of moderate-intensity aerobic training (45–69% VO_2_max), suggesting that exercise performed within this intensity range is effective for improving redox balance.

Several mechanisms have been proposed to explain the concurrent reductions in oxidative stress and increases in antioxidant status observed in prior studies and in the present investigation. In particular, exercise-induced ROS production has been shown to act as a secondary signaling mechanism that upregulates both enzymatic and non-enzymatic antioxidant defense systems, thereby enhancing cellular redox homeostasis and cytoprotective protein expression [7,30]. However, because these mechanistic pathways were not directly assessed in the present study, such interpretations should be considered speculative.

Although some evidence suggests that exercise-induced improvements in oxidative stress can occur independently of weight loss through ROS-mediated signaling and antioxidant adaptation, the majority of available literature emphasizes the importance of reductions in adiposity for sustained improvements in oxidative stress biomarkers [31,32,33]. Excess adipose tissue is strongly associated with elevated ROS production, and reductions in fat mass have been consistently linked to improvements in oxidative stress status. In the present study, greater within-group reductions in adiposity were observed in the HT group; however, given the absence of significant post-intervention between-group differences in oxidative stress biomarkers, these findings suggest that participation in moderate-intensity aerobic exercise, rather than exercise intensity per se, was sufficient to elicit favorable redox adaptations.

In the present study, several possible explanations may account for the greater reduction in %BF observed in the HT group compared to the LT group. One possibility, as suggested by previous studies, is that although both interventions were conducted at moderate intensity with equivalent total energy expenditure, subjects in the HT group may have experienced elevated excess post-exercise oxygen consumption (EPOC) due to increased heart rate and body temperature from each exercise session [34,35]. This elevated EPOC may have contributed to higher total daily energy expenditure. Additionally, increased catecholamine circulation—particularly epinephrine—often associated with higher-intensity exercise, may have further enhanced EPOC and fat oxidation [36,37]. Finally, increased muscle activation for posture and stability during high-intensity treadmill exercise likely contributes further to energy expenditure and fat loss. Carson et al. [38] examined treadmill running with altered forward postural lean and found that greater lean significantly increased metabolic cost (~8%), accompanied by increased activation of hip and thigh extensor muscles, showing how posture contributes to energy expenditure.

Several factors may explain why 8-OHdG and TAS levels appeared similar between the two exercise groups following the intervention, despite the higher intensity and greater fat loss observed in the HT group. One plausible explanation is that the greater reduction in body fat percentage (%BF) in the HT group may have mitigated the potentially increased ROS production associated with higher-intensity exercise, resulting in 8-OHdG and TAS levels comparable to those of the LT group. Additionally, while exercise intensity varied, the total exercise volume was equivalent across both groups, which may have moderated the oxidative response. This aligns with findings by Thirupathi et al. [29], who emphasized that exercise volume, rather than intensity alone, plays a more critical role in modulating oxidative stress and antioxidant capacity. Lastly, as both exercise interventions were conducted within the moderate-intensity range, it is likely that subjects in both groups experienced similar systemic adaptations to oxidative stress. Radak et al. [39] noted that regular aerobic training induces systemic redox adaptations that help maintain redox homeostasis, even under varying exercise intensities. These combined factors may explain the comparable 8-OHdG and TAS outcomes observed in this study, despite apparent differences in exercise intensity and fat loss between the groups.

The present study possesses several notable strengths. Firstly, it was a randomized controlled trial involving 12 weeks of supervised, individualized training conducted in a cardio training facility. In addition, weekly energy expenditure was standardized relative to body weight using METs·h/week, enabling subjects to perform a comparable volume of exercise regardless of individual body weight. These factors contribute to our confidence in the appropriateness and consistency of the exercise intervention.

Nonetheless, this study also has several limitations. Although the sample size was adequately powered (80% power at α = 0.05) to detect changes in abdominal fat mass, it might be insufficient to reliably detect changes in inflammatory cytokines and oxidative stress biomarkers, which are typically characterized by greater biological variability. As a result, the study may have been underpowered to detect modest between-group differences in these biomarkers, raising the possibility of Type II error for intensity-related comparisons.

Secondly, daily dietary intake and physical activity outside of the intervention were not monitored or controlled. Given the strong influence of diet on CRP, adiponectin, and oxidative stress, unmeasured dietary changes or non-intervention physical activity may have contributed to the observed changes in body composition and biomarkers. Accordingly, fat loss and biomarker responses cannot be attributed solely to the prescribed exercise interventions.

Third, several physiological mechanisms discussed in relation to the observed findings, including catecholamine responses, IL-6 signaling, excess post-exercise oxygen consumption, and ROS-mediated redox adaptations, were not directly measured in the present study. Therefore, mechanistic interpretations remain speculative and should be interpreted with caution.

Finally, the study sample was limited to middle-aged, obese Hispanic females. Consequently, the generalizability of the findings to other populations is limited. Although we found no prior studies reporting gender or racial differences in fat loss, inflammation, or oxidative stress responses to varying exercise intensities, to our knowledge, this is the first study to examine the effects of exercise intensity on fat loss, inflammatory cytokines, and redox status specifically in obese Hispanic women. Therefore, our findings should be interpreted within this context.

## 4. Materials and Methods

### 4.1. Subjects

A total of 57 obese Hispanic females aged 34–45 years were recruited from the Hispanic community in the USA. Eligibility criteria included being a non-smoker, premenopausal, obese (BMI > 30 kg/m^2^), physically inactive, and free from chronic diseases. Subjects were excluded if they were on medications that could affect metabolic, cardiovascular, or immune functions or if they had any musculoskeletal limitations. Additionally, subjects who had not menstruated in the past 2 months were excluded. The study was approved by the Institutional Review Board, and all subjects provided written informed consent and completed medical history forms before any study procedures.

### 4.2. Study Design

**Figure 3:**
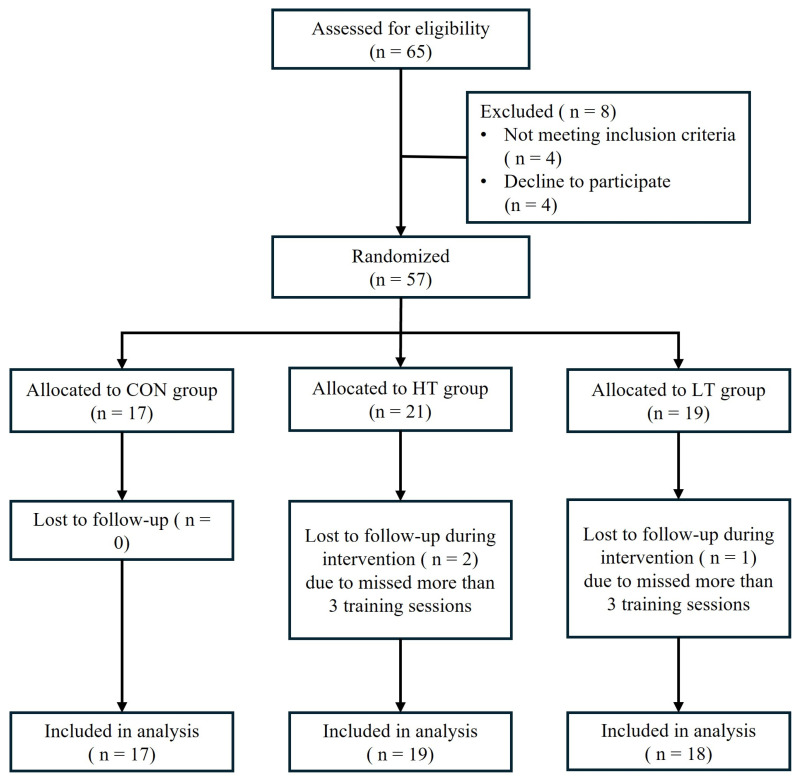
Flow diagram showing participant enrollment, allocation, follow-up, and analysis.

At the initial visit, subjects were randomly assigned to one of following groups: lower-moderate intensity training (LT: n = 19), upper-moderate intensity training (HT: n = 21) or control (CON: n = 17) groups. Assessments were conducted before the intervention (PRE), and after 12 weeks of intervention (POST). Subjects were instructed to maintain their usual diet and to avoid any form of aerobic or anaerobic physical activity outside of the assigned intervention throughout the study. Testing sessions were scheduled between 0800 and 0900 h following a 12 h fast. Each session included anthropometric measurements, body fat and visceral adipose tissue mass assessments, blood sample collection, and a VO_2_max test. Three subjects from the exercise group (one from LT and two from HT) were excluded due to missing more than 3 days of training, resulting in 54 subjects completing the study (18 LT, 19 HT, 17 CON). The flow of participants throughout the study is illustrated in Figure 3.

#### 4.2.1. Anthropometric Measurements

Measurements were performed in duplicate by a trained technician. Height and weight were recorded to the nearest 0.1 cm and 0.1 kg, respectively, with subjects dressed in indoor clothing and without shoes.

#### 4.2.2. Body Composition Assessment

Total and regional body composition was assessed using Dual-energy X-ray Absorptiometry (DXA: GE Lunar Prodigy, Madison, WI, USA) at PRE and POST. This method estimated total lean mass and fat mass in specific regions, including visceral fat. Subjects lay supine on the scanning table for the duration of the scan, which was performed according to standard clinical protocols. They wore a gown and removed all metal objects, such as glasses, jewelry, and cell phones.

#### 4.2.3. VO_2_max Test

The VO_2_max test was conducted two times at pre and post (TrueOne 2400, Parvo Medics, Salt Lake City, UT, USA). The result from the pre-test was used to set the exercise intensity (55% or 70% of VO_2_max) for two exercise groups. VO_2_max was measured during a continuous, progressive treadmill protocol, starting at 4 km/h with speed increments of 0.8 km/h every two minutes.

#### 4.2.4. Blood Collection and Analyses

Five milliliters of venous blood were drawn from an antecubital vein immediately after body fat measurement on each testing day. The blood was centrifuged in serum-separating vacutainer tubes at 1000× *g* for 15 min (Allegra X-15R Refrigerated Centrifuge, Beckman Coulter, Irving, TX, USA). Serum samples were stored at −80 °C until analysis. Enzyme-linked immunosorbent assays (ELISA) were used to measure TNF-α, CRP, adiponectin, TAS, and 8-OHdG. Measurements were conducted with commercial kits (Cayman Chemical Co., Ann Arbor, MI, USA) using a microplate reader (EL 808, BioTek Co., Winooski, VT, USA). The mean intra-assay coefficients of variation (CVs) were 5.9% for TNF-α, 6.3% for CRP, 6.5% for adiponectin, 6.5% for TAS, and 6.8% for 8-OHdG; inter-assay CVs were 6.9%, 7.5%, 7.9%, 6.9%, and 7.3%, respectively.

#### 4.2.5. Exercise Intervention

Subjects in HT and LT groups performed 12 weeks of training at the given exercise intensity (70% and 55% VO_2_max, respectively) using the target heart rate obtained during VO_2_max testing at PRE. Each subject performed exercises and expended 14.2 kcal⋅kg^−1^⋅week^−1^ for the first four weeks (3 sessions per week), 18.9 kcal⋅kg^−1^⋅week^−1^ for weeks 5–8 (4 sessions per week), and 23.7 kcal⋅kg^−1^⋅week^−1^ for last 4 weeks (5 sessions per week). The duration of each exercise session was adjusted according to each subject’s VO_2_-velocity relationship. As each subject’s fitness level improved, the running speed on treadmill was increased in relation to heart rate. In each training session, heart rate was measured telemetrically every 10 min (Polar, Oy, Helsinki, Finland) and average heart rate was calculated. Running speed was adjusted when average heart rate was decreased by more than 5 beats per minute on 2 consecutive training sessions. All exercise sessions were supervised by research assistants. Subjects in control group did not engage in any physical activity during the study. Exercise sessions for both exercise groups were scheduled at the subjects’ convenience, and missed sessions were rescheduled.

### 4.3. Data Analysis

Sample size calculations were performed using G*Power 3.1.0 software, with an alpha level of 0.05, an effect size of 0.40, and a power of 0.80, estimating a required sample size of 15 subjects. Statistical analyses were conducted using SPSS 28 (IBM Corp., Armonk, NY, USA). Two-way repeated-measures multivariate analysis of variance (MANOVA) was employed to analyze changes in body composition, inflammatory cytokines, and oxidative stress markers because all variables met the assumptions of normality (Shapiro–Wilk test) and equal variances (Brown–Forsythe test). Post hoc pairwise comparisons were conducted with Bonferroni correction, with the level of statistical significance set at *p* < 0.05.

## 5. Conclusions

This study demonstrated that 12 weeks of moderate-intensity aerobic exercise, whether performed at upper- or lower-moderate intensity, significantly reduced pro-inflammatory cytokines and improved oxidative stress and antioxidant status in obese Hispanic females. Both lower- and upper-moderate intensity exercise prescriptions were associated with reductions in TNF-α and favorable adaptations in oxidative stress and antioxidant status. Importantly, post-intervention biomarker levels did not differ significantly between exercise groups, indicating that when total exercise volume is matched, moderate-intensity aerobic exercise confers beneficial effects on inflammation and redox balance largely independent of specific intensity prescriptions within the moderate range.

These findings underscore the therapeutic potential of moderate-intensity aerobic exercise as a non-pharmacological strategy to combat chronic low-grade inflammation and oxidative stress in individuals with obesity. For clinical populations unable to tolerate high-intensity exercise, this suggests that low-intensity training such as walking, when sufficient to induce fat loss, may still provide significant benefits in the prevention and management of chronic diseases.

At the same time, the observation that CRP and adiponectin changed significantly only within the upper-moderate intensity group, although not accompanied by significant between-group differences, suggests a potential intensity-related signal that warrants further investigation. Future studies that address the limitations of the present work, including limited statistical power for biomarker outcomes, lack of dietary control, and the absence of direct mechanistic measurements, are needed to clarify whether exercise intensity independently contributes to inflammatory and oxidative stress adaptations beyond the effects of exercise volume and fat loss.

## Figures and Tables

**Table 1:** Physical characteristics of subjects at baseline (PRE) in the CON, HT, and LT groups.

Variables	CON (n = 17)	HT (n = 19)	LT (n = 18)
Age (years)	40.4 ± 3.9	39.6 ± 4.5	39.5 ± 4.4
Height (cm)	162.9 ± 3.3	162.2 ± 5.3	162.6 ± 5.3
Body weight (kg)	90.78 ± 16.08	89.61 ± 17.01	88.91 ± 15.45
BMI (kg/m^2)^	34.20 ± 5.85	33.83 ± 5.70	33.75 ± 5.40
% Body fat	42.38 ± 5.68	42.33 ± 5.84	42.02 ± 4.66
VAT (kg)	1.74 ± 0.55	1.72 ± 0.53	1.74 ± 0.38

Values are means ± SD. CON: Control group, HT: Upper-moderate intensity group, LT: Lower-moderate intensity group, BMI: Body mass index, VAT: Visceral adipose tissue mass.

**Table 2:** Changes in body composition over 12 weeks in CON, HT, and LT groups.

Variable	Group	PRE	POST	Δ Change	*p* (Within)
Body Weight (kg)	CON	90.78 ± 16.08	90.74 ± 16.62	−0.04	0.940
	HT	89.61 ±17.01	85.97 ± 16.65	−3.64	<0.001 **
	LT	88.91 ± 15.45	86.59 ± 14.13	−2.31	<0.001 **
BMI (kg/m^2^)	CON	34.20 ± 5.85	34.18 ± 6.07	−0.01	0.935
	HT	33.83 ± 5.70	32.46 ± 5.65	−1.37	<0.001 **
	LT	33.75 ± 5.40	32.88 ± 4.93	−0.87	<0.001 **
Body Fat (%)	CON	42.38 ± 5.68	42.73 ± 5.75	+0.35	0.345
	HT	42.33 ± 5.84	37.36 ± 4.83	−4.97	<0.001 **
	LT	42.02 ± 4.66	38.61 ± 4.19	−3.41	<0.001 **
VAT (kg)	CON	1.74 ± 0.55	1.75 ± 0.155	+0.007	0.853
	HT	1.72 ± 0.53	1.45 ± 0.35	−0.268	<0.001 **
	LT	1.74 ± 0.38	1.66 ± 0.35	−0.079	0.037 *

Values are means ± SD. CON: control group, HT: Upper-moderate intensity group, LT: Lower-moderate intensity group, BMI: Body mass Index, VAT: Visceral adipose tissue mass. * significantly different from PRE within group (*p* < 0.05); ** significantly different from PRE within group (*p* < 0.01).

## Data Availability

The datasets used or analyzed during the current study are available from the corresponding author upon reasonable request.

## Abbreviations

The following abbreviations are used in this manuscript:

TNF-α	Tumor necrosis factor-alpha
CRP	C-reactive protein
TAS	Total antioxidant status
8-OHdG	8-Hydroxy-2′-deoxyguanosine
%BF	Body fat percentage
VAT	Visceral adipose tissue
DXA	Dual-energy X-ray absorptiometry
ROS	Reactive oxygen species
OS	Oxidative stress
LT	Lower-moderate intensity
HT	Upper-moderate intensity
CON	Control
PRE	Before 12 weeks of intervention
POST	After 12 weeks of intervention
BMI	Body mass index
